# Evaluate Laser Needle Effect on Blood Perfusion Signals of Contralateral Hegu Acupoint with Wavelet Analysis

**DOI:** 10.1155/2012/103729

**Published:** 2012-09-13

**Authors:** Guangjun Wang, Yuying Tian, Shuyong Jia, Gerhard Litscher, Weibo Zhang

**Affiliations:** ^1^Institute of Acupuncture and Moxibustion, China Academy of Chinese Medical Sciences, Beijing 100700, China; ^2^Stronach Research Unit for Complementary and Integrative Laser Medicine, Research Unit of Biomedical Engineering in Anesthesia and Intensive Care Medicine, and TCM Research Center Graz, Medical University of Graz, Auenbruggerplatz 29, 8036 Graz, Austria

## Abstract

Our previous studies suggested that the MBF in contralateral Hegu acupoint (IL4) increased after ipsilateral Hegu acupoint was stimulated with manual acupuncture. In this study, twenty-eight (28) healthy volunteers were recruited and were randomly divided into Hegu acupoint stimulation group and Non-Hegu stimulation group. All subjects received the same model stimulation of the laser needle for 30 min in right Hegu acupoint and Non-Hegu acupoint, respectively. MBF of left LI4 was measured by the laser Doppler perfusion imaging system. The original data dealt with morlet wavelet analysis and the average amplitude and power spectral density of different frequency intervals was acquired. The results indicated that right Hegu stimulation with the laser needle might result in the increase of left Hegu acupoint MBF. 40 min later after ceased stimulation, the MBF is still increasing significantly, whereas the MBF has no significantly change in Non-Hegu stimulation group. The wavelet analysis result suggested that compared to Non-Hegu stimulation, stimulated to right Hegu acupoint might result in the increase of average amplitude in frequency intervals of 0.0095–0.02 Hz, 0.02–0.06 Hz, and 0.06–0.15 Hz, which might be influenced by the endothelial, neurogenic, and the intrinsic myogenic activity of the vessel wall, respectively.

## 1. Introduction 

Acupuncture has been widely used to the treatment of diseases in clinical practice at least for 2000 years [1]. According to the principles of *Huang Di Nei Jing Su Wen *[2], acupuncture effects might be related to the appropriate acupoints selection during the treatment. However, many researchers firmly believe that placebo effect may be the best explanation for acupuncture [3]. A large number of clinical trials have reported that true acupuncture is superior to usual care, but is not significantly better than sham acupuncture, findings apparently at odds with acupoint specificity [4]. On the contrary, many researchers pointed out that distribution of blood perfusion has specificity in acupoint and meridians compared with no acupoint or no meridian areas. Assuming the acupoint was stimulated adequately, the blood flow of this point increased whereas the blood flow of non-acupoint only changed slightly by the same stimulation [5]. Needling the LI4 significantly increased perfusion at Hegu acupoint but not at nearby nonacupoint [6]. 

Our previous studies have shown that thermostimulation could result in an increase of blood perfusion not only in the local area, but also in the same area of contralateral side. This phenomenon can be observed both in the upper limb and lower limb, but not around the periumbilicus area. It indicated that bilateral blood perfusion of same area might be special relationships. We have reported that stimulation on side LI4 by the manual acupuncture might result in the increase of blood perfusion on the contralateral LI4, which indicated that there might be intrinsic correlation between contra and ipsilateral parts [7, 8]. Our work was supported by Kubo et al. [9]. Furthermore, we explored the MBF of bilateral LI4 with system identification algorithm and found that the LI4 has lateralized specificity. Stimulating right LI4 by manual acupuncture might produce the more forcefull amplificatory effect than stimulating left LI4 [7, 8]. 

Recently, the laser needle as an alternative method of manual acupuncture was used in many studies because it is effective not only as a treatment method, but also as a research tool [10–12]. It can simulate traditional acupuncture while reduceing the stress effect resulted from the mental needle. So in this study, we select laser needle as stimulation tool and observe the contralateral effect which resulted from laser acupuncture.

## 2. Methods

### 2.1. Ethics Statement

This study was reviewed and approved by the Institutional Review Board of the Institute of Acupuncture and Moxibustion, China Academy of Chinese Medical Sciences. Each study participant read and signed an informed consent form.

### 2.2. Subjects

Twenty-eight (28) healthy volunteers were recruited in this study. All subjects were students from the China Academy of Chinese Medical Sciences and Beijing University of TCM and all of them had no history of diseases and had not taken any medicine in the past six months before study. The detail information of subjects is shown in Table 1. Each subject provided informed consent and had an adequate understanding of the procedure and purpose of this study. 

### 2.3. Protocol for MBF Measurement

**Figure 1:**
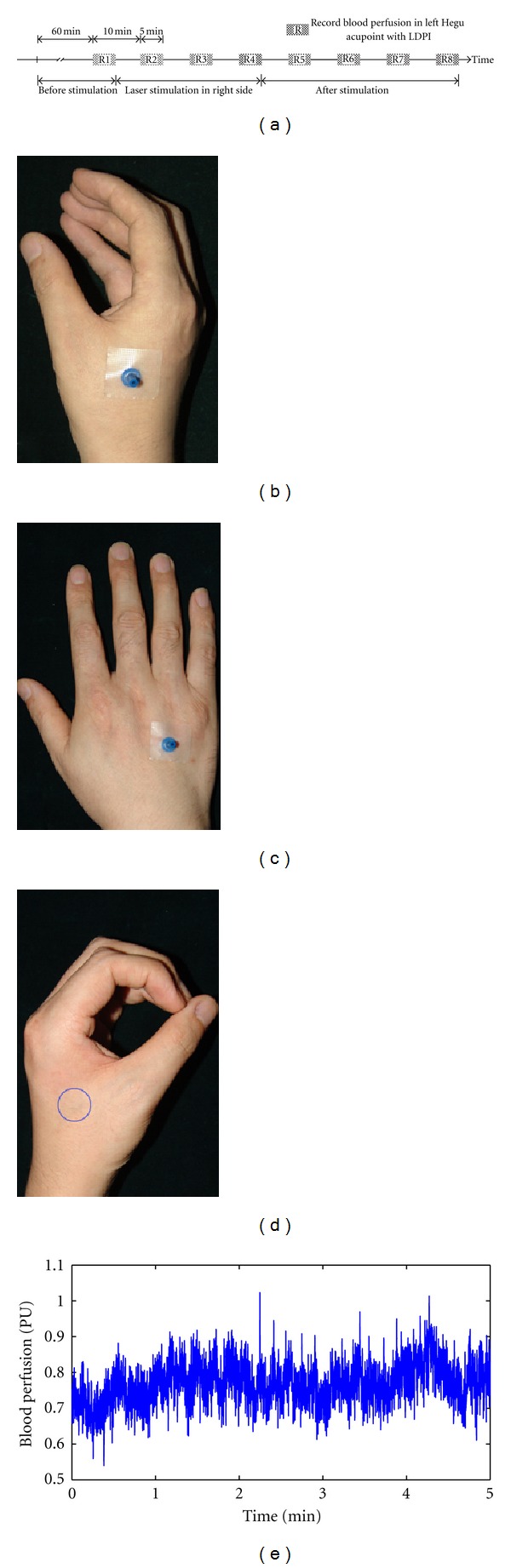
Illustration of the study design. Measurement and stimulation procedures (a); Hegu acupoint stimulation site (b); Non-Hegu stimulation site (c); measurement site of left Hegu acupoint marked as blue circle (d); the original signal of blood flux (e).

Before arrival to the laboratory, subjects were placed in a temperature-controlled room (24–26°C) as a resting state for 60 minutes. Measurements of skin blood perfusion in left LI4 (as shown in Figure 1(d)) were carried out using laser doppler perfusion imager (LDPI, PeriScan PIM II system, Perimed AB, Sweden). Before recording, left hands were immobilized with a cylindrical object to ensure positioning. The measurement parameters were as follows: NR model; duplex mode; one measurement site; 100 Hz sample rate. Measurement was carried out every 5 minutes over a total of 80 min (as shown in Figure 1(a)). During the experiment, the laboratory room was kept in dark light condition and the protocol for measurement operation was abided strictly. In recording process, ensuring the laser beam was inside the selected area marked by a blue circle (Figure 1(d)). MBF (symbolized as ${\overset{-}{R}}_{i}\;\;(i=1,2,3,\ldots ,8)$) was defined as mean of blood flux in 5 min. The change of MBF was defined as $\left({\overset{-}{R}}_{i}-{\overset{-}{R}}_{1})/{\overset{-}{R}}_{1}\;\;(i=1,2,3,\ldots ,8\right)$. 

### 2.4. Laser Acupuncture Protocol

For acupuncture, the position of LI4 was confirmed according to the previous studies [13, 14], as shown in Figure 1(b). Non-Hegu position was defined as between thirty-four metacarpal, in the same plane of LI4 (Figure 1(c)). In H-S group and N-HS group, the laser needle was attached to right LI4 and Non-Hegu position, respectively. Just in the H-S group, the appliance was switched on, and the lasers were activated. The radiation model is continuous radiation (CW) and radiation power is ca. 50 mW. The radiation time keeps 30 min continuously (Figure 1(a)).

### 2.5. Data Analysis

For every record epoch of 5 min, the MBF was calculated to elucidate the effect of contralateral laser needle stimulation. According to previous studies, microvascular blood perfusion signal can be separated into five components, which were influenced by the endothelial activity, the neurogenic activity, the intrinsic myogenic activity, the respiration, and the heartbeat, respectively [6, 15–17]. Morlet mother wavelet was applied to improve the low-frequency resolution. In frequency domain, five characteristic frequency components were separated by 0.0095–0.02, 0.02–0.06, 0.06–0.15, 0.15–0.4, and 0.4–1.6 Hz frequency bands (symbolized as FR1 to FR5, resp.) and the average amplitude (symbolized as ${\overset{-}{A}}_{i}\;\;(i=1,2,3,\ldots ,8)$) in every frequency band was calculated with morlet wavelet analysis method [6]. The change of average amplitude was defined as $\left({\overset{-}{A}}_{i}-{\overset{-}{A}}_{1})/{\overset{-}{A}}_{1}\;\;(i=1,2,3,\ldots ,8\right)$. The energy distribution was symbolized as *P*_*i*_  (*i* = 1,2, 3,…, 8) in every frequency band, and for every FR, the change of energy distribution from R1 to R8 which defined as (*P*_*i*_ − *P*_1_)/*P*_1_  (*i* = 1,2, 3,…, 8) was also calculated. All signal processing was performed with MATLAB (MathWorks, Natick, MA, USA). Two-tailed paired *t*-test and independent *t*-test were used to verify the statistical significance. Differences were considered significant when *P* < 0.05.

## 3. Results 

### 3.1. MBF Response

**Figure 2:**
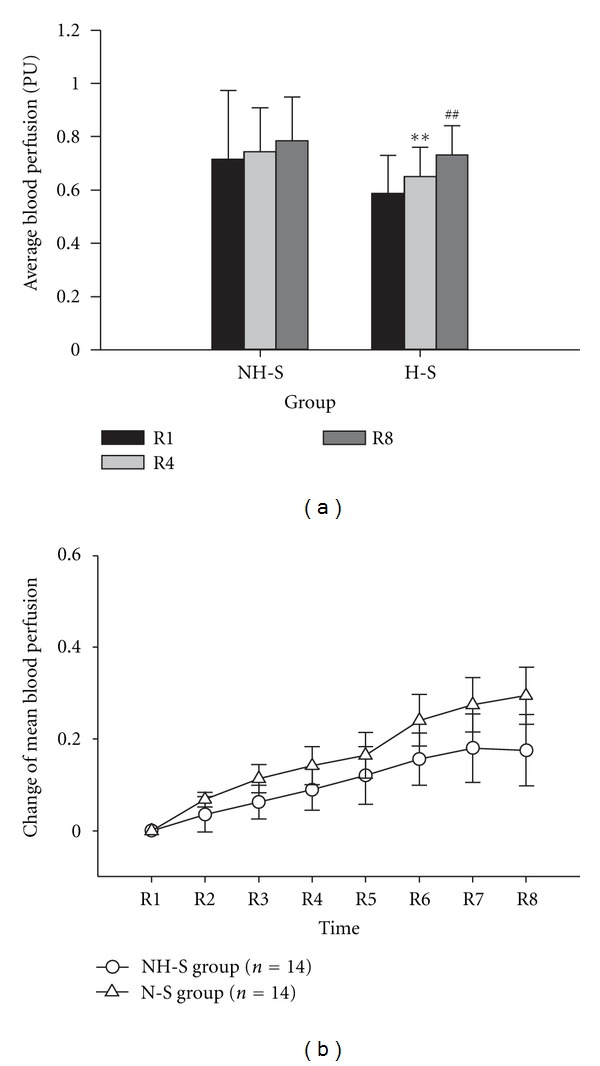
Change of MBF in left Hegu acupoint. All data expressed as Mean ± SEM. NH-S: Non-Hegu stimulation; H-S: Hegu acupoint stimulation; (a) shows the average blood flux in NH-S group and H-S group, **P < 0.01, R1 versus R4; ^##^ *P* < 0.01, R4 versus R8. (b) shows the change of MBF in different time period, which defined as $\left({\overset{-}{R}}_{i}-{\overset{-}{R}}_{1})/{\overset{-}{R}}_{1}\;\;(i=1,2,3,\ldots ,8\right)$. In every time, the change of MBF between two group has not significant difference (*P* > 0.05).

The MBF in different time was compared both in H-S group and NH-S Group (as shown in Table 2 and Figure 2). In H-S group, the MBF of left Hegu acupoint was significantly increased after laser stimulation (*P* < 0.01, R1 versus R4, paired *t*-test). 40 min later after ceased laser stimulation, the MBF was significantly increased (*P* < 0.01, R4 versus R8, paired *t*-test). However, in NH-S group, there is no significant increase of MBF both in R1 versus R4 and in R4 versus R8 (Table 2 and Figure 2(a)). In every time point, there is no significant difference in change of MBF between H-S and NH-S groups (as shown in Table 3 and Figure 2(b)). 

### 3.2. LDF Flux Spectra

**Figure 3:**
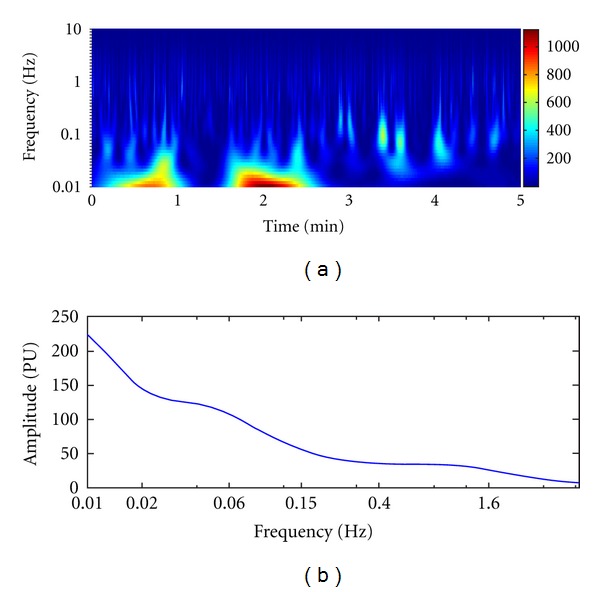
Morlet wavelet analysis result of original signal. (a) shows the time-frequency relationship; (b) shows the frequency-amplitude relationship.

**Figure 4:**
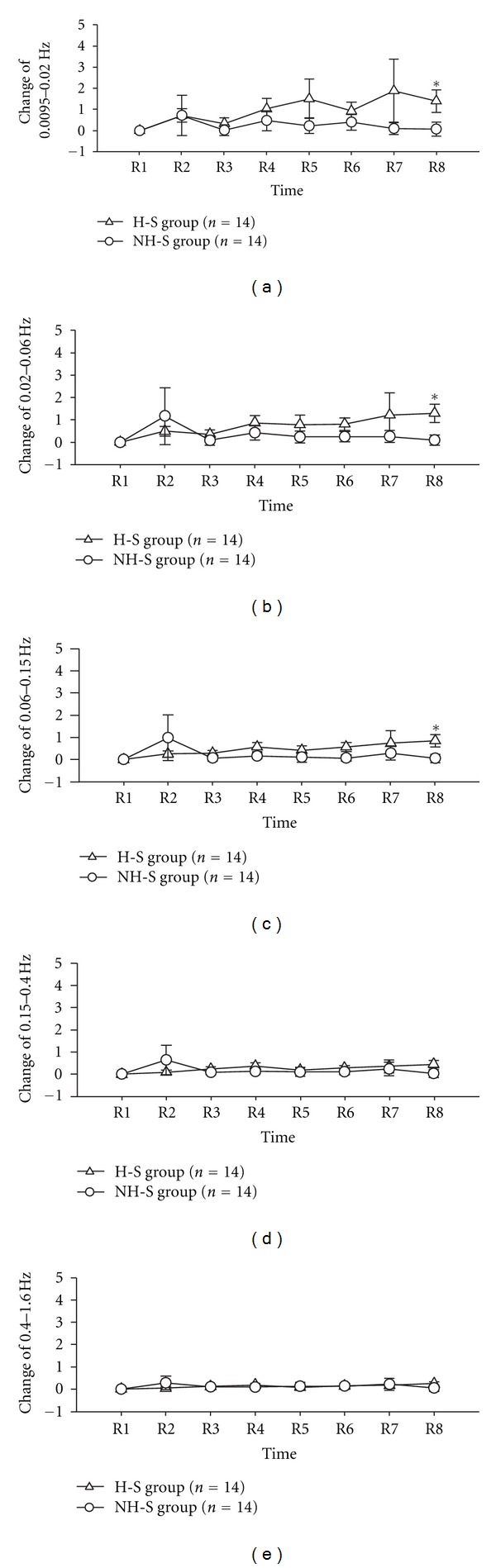
Change of average amplitude in left Hegu acupoint. The change of average amplitude is defined as $\left({\overset{-}{A}}_{i}-{\overset{-}{A}}_{1})/{\overset{-}{A}}_{1\;\;}(i=1,2,3,\ldots ,8\right)$. All data expressed as Mean ± SEM. NH-S: Non-Hegu stimulation; H-S: Hegu acupoint stimulation; (a) shows the frequency interval of 0.0095–0.02 Hz; (b) shows the frequency interval of 0.02–0.06 Hz; (c) shows the frequency interval of 0.06–0.15 Hz; (d) shows the frequency interval of 0.15–0.4 Hz; (e) shows the frequency interval of 0.4–1.6 Hz; *P < 0.05, H-S versus NH-S.

**Figure 5:**
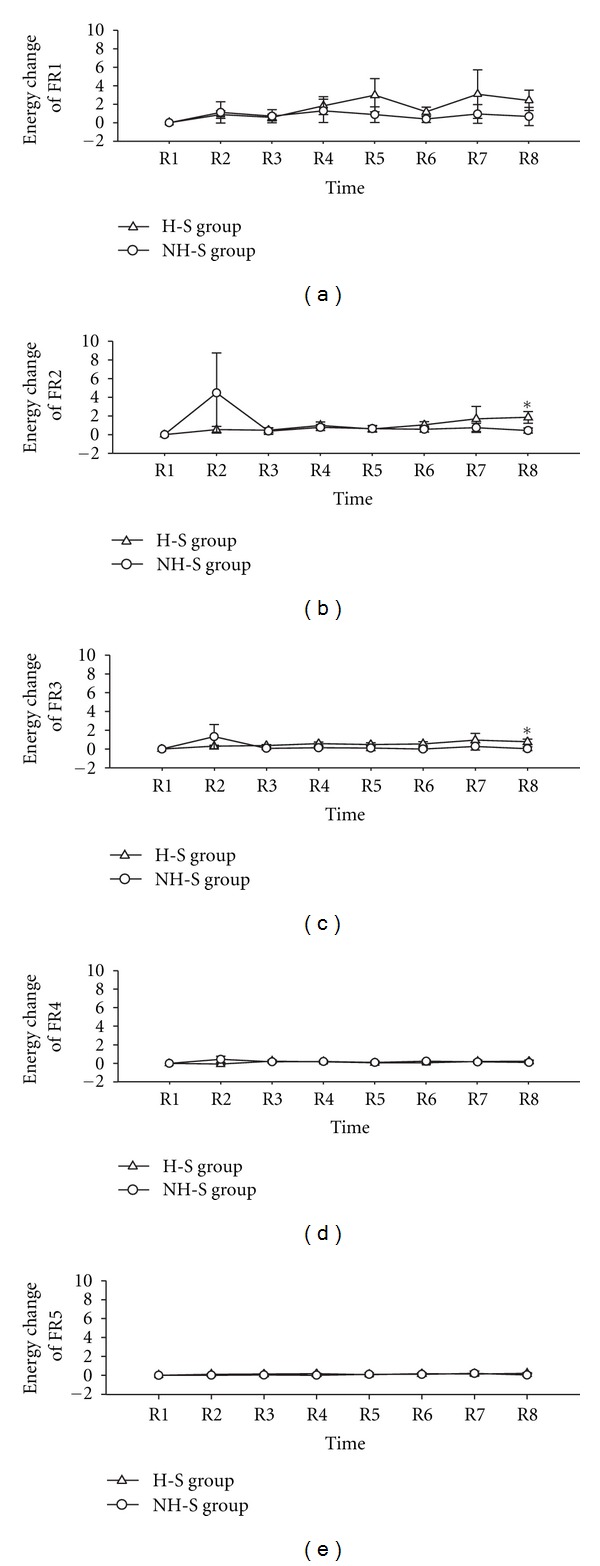
Change of energy distribution in left Hegu acupoint. The energy distribution in every frequency band was symbolized as *P*_*i*_  (*i* = 1,2, 3,…, 8), and for every FR, the change of energy distribution from R1 to R8 was defined as (*P*_*i*_ − *P*_1_)/*P*_1_  (*i* = 1,2, 3,…, 8). All data expressed as Mean ± SEM. NH-S: Non-Hegu stimulation; H-S: Hegu acupoint stimulation; (a) shows the frequency interval of 0.0095–0.02 Hz; (b) shows the frequency interval of 0.02–0.06 Hz; (c) shows the frequency interval of 0.06–0.15 Hz; (d) shows the frequency interval of 0.15–0.4 Hz; (e) shows the frequency interval of 0.4–1.6 Hz; *P < 0.05, H-S versus NH-S.

The result of wavelet analysis is shown in Table 4 and Figure 3. The time-frequency relationship is shown in Figure 3(a), and the frequency-amplitude relationship is shown in Figure 3(b). Change of average amplitude in the different frequency intervals following laser stimulation at the different time points is compared in Figure 4. 40 min later after ceased laser stimulation, the change of average amplitude in frequency intervals of FR1 (0.0095–0.02 Hz), FR2 (0.02–0.06), and FR3 (0.06–0.15) was significantly different between H-S group and NH-S group (*P* < 0.05, two-tailed *t*-test), as shown in Table 4 and Figures 4(a), 4(b), and 4(c). In other time point and other frequency intervals, there are no significant differences between H-S group and NH-S group (as shown in Table 4 and Figures 4(d) and 4(e)). The result of energy distribution was shown in Table 5 and Figure 5. It can be found that the change of energy distribution in frequency intervals of FR2 (0.02–0.06) and FR3 (0.06–0.15) was significantly different between H-S group and NH-S group (*P* < 0.05, two-tailed *t*-test). 

## 4. Discussion

Up to now, it is difficult to evaluate the activation of acupoints, and as a result, it is also difficult to analyse the specificity of acupoints after meridians stimulated. Recently, more and more attention has been focused on the relationship of acupuncture and circulation [18–20]. In Traditional Chinese Medical (TCM) theory, one of the definitive causes of acupuncture effect is the special sensation in local acupoint after stimulation, which might be related to the blood perfusion changes in acupoints or meridians [13]. According to the previous study, the MBF was larger at the acupoints than in their surrounding tissues, which indicates that the MBF can be used as an index for discriminating differences in the microcirculatory conditions between acupoints and their surrounding tissues [21]. It has also been shown that acupuncture cannot only increase general circulation [22] and circulation in specific organs [23], but change the skin microcirculation as well [14, 19, 24, 25]. When an acupoint was stimulated adequately, the blood perfusion of this point continued to increase, whereas the blood perfusion of non-acupoint only changed slightly by the same acupuncture stimulation [5]. These results indicated that the blood perfusion in acupoints can be recommended as a candidate index for acupuncture effect evaluation.

Recently, Laser Doppler flowmetry (LDF) is widely used for monitoring the microcirculation due to its advantages of a good frequency response and is suited for noninvasive investigations of microvascular responses to acupuncture [18, 26]. In this study, the result suggested that stimulated right LI4 with laser needle and the BMF in left Hegu acupoint increased significantly, which brings into correspondence with our previous study [27, 28]. In this study, when laser stimulation was ceased, the MBF increased in the symmetric area, while Non-Hegu acupoint stimulation has no effect in left LI4, which suggested that the laser needle effect might have area specificity. 

Spectral analysis of LDF signals reveals that blood-flow oscillations at frequencies from 0.009 to 1.6 Hz might reflect various physiological rhythms [17, 29]. In this study, LDF signals spectral were analyzed with morlet wavelet analysis [6, 14, 19]. The results indicated that right LI4 stimulation with laser needle just affect the amplitude of FR1, FR2, and FR3, which can be influenced by the endothelial, neurogenic, and the intrinsic myogenic activity of vascular smooth muscle, respectively. After analyzed energy distribution, we found from R1 to R8 that the change of energy in different FRs has the same change trend as amplitude. In accord with the suggestion that the skin microvasculature mirrors the vascular function of other parts of the body [30–32], we also can suppose that the vascular function of left LI4 is the mirror of the right LI4, or partly the mirror of right LI4. From our study, the FR1, FR2, and FR3 take part in the action of laser stimulation, which means that the endothelial activity, the neurogenic activity, and the intrinsic myogenic activity might be the candidates of the underlying physiological mechanism of this function mirror. Compared to NH-S group, the laser needle effect observed 80 min later after laser stimulation, which suggested that laser stimulation in special acupoint might have resulted in slow and complicated reaction and this mirror function is not happened at once after stimulation. 

## Figures and Tables

**Table 1:** Subjects' gender composition, average age, and body mass index.

Group	*n*	Gender (female/male)	Age (years, mean ± SD)	Body mass index (mean ± SD)
H-S group	14	13/1	24.36 ± 2.06	21.35 ± 3.19
NH-S group	14	11/3	24.43 ± 1.74	20.61 ± 1.66

H-S: Hegu acupoint stimulation; NH-S: Non-Hegu acupoint stimulation.

**Table 2:** MBF in different group (PU, Mean ± SEM).

Record point	H-S group	NH-S group
R1	0.587 ± 0.041	0.716 ± 0.071
R4	0.651 ± 0.031^∗∗^	0.744 ± 0.046
R8	0.731 ± 0.032^##^	0.784 ± 0.046

^∗∗^ *P* < 0.01 R4 versus R1; ^##^ *P* < 0.01 R8 versus R4.

**Table 3:** Change of MBF in different group (Mean ± SEM).

Record point	H-S group	NH-S group
R1	0.000 ± 0.000	0.000 ± 0.000
R2	0.068 ± 0.016	0.035 ± 0.038
R3	0.113 ± 0.031	0.062 ± 0.037
R4	0.142 ± 0.042	0.089 ± 0.045
R5	0.165 ± 0.050	0.121 ± 0.063
R6	0.241 ± 0.056	0.156 ± 0.057
R7	0.275 ± 0.059	0.180 ± 0.075
R8	0.294 ± 0.062	0.175 ± 0.077

Change of MBF in different time point is defined as $\left({\overset{-}{R}}_{i}-{\overset{-}{R}}_{1}\right)/{\overset{-}{R}}_{1}(i=1,2,3,\ldots ,8)$.

*P* > 0.05.

**Table 4:** Change of average amplitude in different frequency intervals (Mean ± SEM).

Frequency intervals	Record point	H-S group	NH-S group
FR1	R1	0.000 ± 0.000	0.000 ± 0.000
	R2	0.722 ± 0.323	0.722 ± 0.947
	R3	0.338 ± 0.266	0.029 ± 0.253
	R4	1.032 ± 0.480	0.476 ± 0.475
	R5	1.505 ± 0.928	0.234 ± 0.374
	R6	0.923 ± 0.426	0.398 ± 0.369
	R7	1.886 ± 1.495	0.105 ± 0.280
	R8	1.399 ± 0.543^∗^	0.076 ± 0.329

FR2	R1	0.000 ± 0.000	0.000 ± 0.000
	R2	0.498 ± 0.225	1.174 ± 1.267
	R3	0.358 ± 0.201	0.086 ± 0.213
	R4	0.875 ± 0.320	0.422 ± 0.332
	R5	0.791 ± 0.432	0.241 ± 0.273
	R6	0.808 ± 0.273	0.255 ± 0.228
	R7	1.225 ± 0.975	0.265 ± 0.259
	R8	1.293 ± 0.417^∗^	0.103 ± 0.222

FR3	R1	0.000 ± 0.000	0.000 ± 0.000
	R2	0.253 ± 0.134	0.989 ± 1.031
	R3	0.296 ± 0.126	0.073 ± 0.131
	R4	0.577 ± 0.206	0.165 ± 0.141
	R5	0.423 ± 0.203	0.115 ± 0.231
	R6	0.567 ± 0.195	0.074 ± 0.145
	R7	0.737 ± 0.580	0.295 ± 0.324
	R8	0.853 ± 0.276^∗^	0.056 ± 0.192

FR4	R1	0.000 ± 0.000	0.000 ± 0.000
	R2	0.089 ± 0.090	0.643 ± 0.669
	R3	0.234 ± 0.097	0.086 ± 0.114
	R4	0.369 ± 0.145	0.124 ± 0.114
	R5	0.187 ± 0.106	0.101 ± 0.206
	R6	0.281 ± 0.111	0.109 ± 0.135
	R7	0.356 ± 0.293	0.242 ± 0.298
	R8	0.455 ± 0.168	0.035 ± 0.169

FR5	R1	0.000 ± 0.000	0.000 ± 0.000
	R2	0.051 ± 0.043	0.282 ± 0.298
	R3	0.126 ± 0.050	0.101 ± 0.112
	R4	0.193 ± 0.065	0.098 ± 0.104
	R5	0.086 ± 0.057	0.133 ± 0.184
	R6	0.148 ± 0.051	0.141 ± 0.127
	R7	0.175 ± 0.135	0.223 ± 0.263
	R8	0.251 ± 0.086	0.051 ± 0.146

^∗^ *P* < 0.05, H-S group versus NH-S group; The change of average amplitude is defined as $\left({\overset{-}{A}}_{i}-{\overset{-}{A}}_{1}\right)/{\overset{-}{A}}_{1}(i=1,2,3,\ldots ,8)$.

**Table 5:** Change of energy distribution in different frequency intervals (Mean ± SEM).

Frequency intervals	Record point	H-S group	NH-S group
FR1	R1	0.000 ± 0.000	0.000 ± 0.000
	R2	0.877 ± 0.420	1.104 ± 1.162
	R3	0.558 ± 0.368	0.715 ± 0.706
	R4	1.831 ± 0.956	1.280 ± 1.268
	R5	2.965 ± 1.798	0.870 ± 0.854
	R6	1.169 ± 0.506	0.394 ± 0.357
	R7	3.086 ± 2.635	0.937 ± 1.022
	R8	2.416 ± 1.095	0.679 ± 0.988

FR2	R1	0.000 ± 0.000	0.000 ± 0.000
	R2	0.527 ± 0.362	4.479 ± 4.244
	R3	0.463 ± 0.232	0.384 ± 0.342
	R4	0.981 ± 0.352	0.767 ± 0.322
	R5	0.596 ± 0.331	0.633 ± 0.355
	R6	1.046 ± 0.358	0.576 ± 0.321
	R7	1.678 ± 1.347	0.728 ± 0.489
	R8	1.850 ± 0.619^∗^	0.438 ± 0.262

FR3	R1	0.000 ± 0.000	0.000 ± 0.000
	R2	0.293 ± 0.121	1.305 ± 1.302
	R3	0.357 ± 0.127	0.080 ± 0.152
	R4	0.555 ± 0.179	0.126 ± 0.138
	R5	0.481 ± 0.179	0.092 ± 0.268
	R6	0.543 ± 0.223	−0.006 ± 0.152
	R7	0.934 ± 0.709	0.279 ± 0.386
	R8	0.766 ± 0.290^∗^	0.028 ± 0.208

FR4	R1	0.000 ± 0.000	0.000 ± 0.000
	R2	−0.093 ± 0.082	0.422 ± 0.341
	R3	0.233 ± 0.141	0.154 ± 0.105
	R4	0.171 ± 0.109	0.198 ± 0.114
	R5	0.045 ± 0.102	0.082 ± 0.129
	R6	0.072 ± 0.108	0.209 ± 0.146
	R7	0.195 ± 0.167	0.139 ± 0.103
	R8	0.209 ± 0.167	0.102 ± 0.145

FR5	R1	0.000 ± 0.000	0.000 ± 0.000
	R2	0.098 ± 0.035	0.010 ± 0.088
	R3	0.124 ± 0.036	0.039 ± 0.126
	R4	0.154 ± 0.047	0.004 ± 0.098
	R5	0.078 ± 0.035	0.093 ± 0.177
	R6	0.163 ± 0.044	0.101 ± 0.144
	R7	0.138 ± 0.063	0.185 ± 0.248
	R8	0.201 ± 0.054	0.019 ± 0.127

^∗^ *P* < 0.05, H-S group versus NH-S group.
